# Impact of Thyroid Transcription Factor‐1 Expression on Outcomes of Platinum‐Based Chemoimmunotherapy in Advanced or Recurrent Non‐Squamous Non‐Small Cell Lung Cancer

**DOI:** 10.1111/1759-7714.70335

**Published:** 2026-06-30

**Authors:** Ryohei Kamada, Hiroshi Yokouchi, Shohei Mizobuchi, Ken Kuwahara, Yasushi Mizukami, Hidenori Mizugaki, Noriyuki Yamada, Hajime Asahina, Hirofumi Adachi, Yoshihiro Matsuno, Satoshi Oizumi

**Affiliations:** ^1^ Department of Respiratory Medicine NHO Hokkaido Cancer Center Sapporo Japan; ^2^ Department of Pathology NHO Hokkaido Cancer Center Sapporo Japan; ^3^ Department of Thoracic Surgery NHO Hokkaido Cancer Center Sapporo Japan

**Keywords:** chemoimmunotherapy, immune‐related adverse events, non‐small cell lung carcinoma, thyroid transcription factor‐1

## Abstract

**Background:**

Thyroid transcription factor‐1 (TTF‐1) is a diagnostic biomarker in non‐squamous (non‐sq) non‐small cell lung cancer (NSCLC). TTF‐1 negativity has been associated with poor outcomes following immunotherapy and pemetrexed‐based chemotherapy in advanced non‐sq NSCLC. However, the impact of TTF‐1 expression on chemoimmunotherapy efficacy regimens remains unclear.

**Methods:**

We retrospectively analyzed patients with advanced or recurrent non‐sq NSCLC who received first‐line platinum and pemetrexed plus pembrolizumab (Platinum/PEM/Pembro) or carboplatin and nab‐paclitaxel plus atezolizumab (CBDCA/nab‐PTX/Atezo) between March 2019 and May 2025 at our institution.

**Results:**

Among 67 patients, 44 were TTF‐1 positive and 23 were TTF‐1 negative. In the overall cohort, TTF‐1‐positive patients had significantly longer progression‐free survival (PFS) and overall survival (OS) than TTF‐1‐negative patients. In the Platinum/PEM/Pembro group, PFS and OS did not differ by TTF‐1 status (median PFS: 8.5 vs. 7.0 months; hazard ratio [HR], 0.85; 95% confidence interval [CI], 0.39–1.86, and median OS: 23.7 vs. 26.1 months; HR, 0.93; 95% CI, 0.36–2.35). In univariate analysis of the overall population, TTF‐1 expression and performance status (PS) were significantly associated with PFS, while TTF‐1 expression, PS, and histological type were significantly associated with OS. The Platinum/PEM/Pembro group had a higher relative dose intensity of concomitant chemotherapy, a greater median number of immune checkpoint inhibitor cycles, and a longer median treatment duration. Immune‐related adverse events (irAEs) were more frequent with Platinum/PEM/Pembro (50.0% vs. 17.4%, *p* = 0.0164).

**Conclusions:**

Platinum/PEM/Pembro may be a therapeutic option for TTF‐1‐negative non‐sq NSCLC with good PS and adenocarcinoma histology, although careful management of irAE is required.

## Graphical abstract

Among thyroid transcription factor‐1 (TTF‐1) negative non‐squamous (non‐sq) NSCLC patients, platinum and pemetrexed plus pembrolizumab showed comparable and preferable efficacy in terms of progression‐free survival and overall survival to that observed in TTF‐1‐positive patients. This regimen may be a therapeutic option for TTF‐1‐negative non‐sq NSCLC with good performance status and adenocarcinoma histology, although careful management of immune‐related adverse event is required.

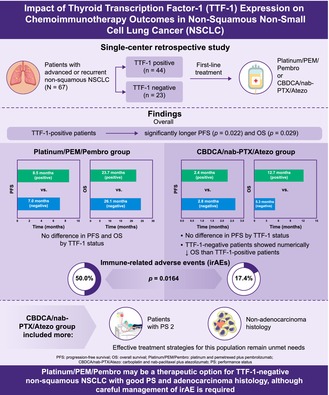

## 1 Introduction

Lung cancer remains the leading cause of cancer‐related mortality worldwide [1]. Therapeutic outcomes for advanced lung cancer have improved substantially with the clinical development of immune checkpoint inhibitors (ICIs) [2, 3, 4]. In addition, combination strategies such as chemoimmunotherapy have been established as one of the standard first‐line treatments for patients with advanced non‐small cell lung cancer (NSCLC), largely replacing conventional platinum‐based chemotherapy alone [5, 6].

Thyroid transcription factor‐1 (TTF‐1) is a nuclear transcription factor involved in the regulation of surfactant protein expression and plays a critical role in lung development and differentiation [7, 8, 9]. TTF‐1 is expressed in approximately 69%–80% of non‐squamous (non‐sq) NSCLC cases and is widely used in routine pathological practice to determine histological subtype and distinguish primary lung adenocarcinoma from metastatic adenocarcinoma of other origins [10, 11, 12, 13]. In addition to its diagnostic utility, TTF‐1 contributes to tumor cell differentiation and suppresses metastatic potential in lung adenocarcinoma. Loss of TTF‐1 expression has been associated with dedifferentiation, enhanced tumor seeding ability, and increased metastatic behavior [14, 15].

Several studies have reported inferior responses to pemetrexed‐based chemotherapy in patients with TTF‐1‐negative NSCLC [16, 17, 18, 19]. Recent meta‐analyses further demonstrated that TTF‐1 negativity is associated with poorer prognosis in patients treated with immunotherapy or chemoimmunotherapy [20]. However, in these studies, pemetrexed‐based chemoimmunotherapy constituted the majority of cases, and data evaluating non‐pemetrexed‐based chemoimmunotherapy regimens according to TTF‐1 expression were limited. Against this background, Shiraishi et al. conducted a phase II trial evaluating carboplatin and nab‐paclitaxel plus atezolizumab (CBDCA/nab‐PTX/Atezo) in patients with TTF‐1‐negative advanced non‐sq NSCLC. Nevertheless, the study failed to meet its primary endpoint of progression‐free survival (median 4.9 months) [21], and thus a taxane‐based combination regimen has not been established as a standard treatment in this population. Accordingly, the impact of TTF‐1 expression on the efficacy of different chemoimmunotherapy regimens in this setting remains incompletely defined.

The KEYNOTE‐189 [5] and IMpower130 [6] trials established platinum plus pemetrexed with pembrolizumab (Platinum/PEM/Pembro) and CBDCA/nab‐PTX/Atezo, respectively, as standard treatments for advanced or recurrent non‐sq NSCLC. We therefore investigated whether TTF‐1 expression is associated with the therapeutic efficacy of these two regimens.

## 2 Methods

### 2.1 Study Design and Patient Selection

In this single‐center retrospective study, we analyzed 67 patients with non‐sq NSCLC, available TTF‐1 expression status, and stage III disease that was not amenable to surgery or definitive radiotherapy, stage IV disease, or postoperative recurrent disease. The patients had started combination treatment with Platinum/PEM/Pembro or CBDCA/nab‐PTX/Atezo in the first‐line setting between March 2019 and May 2025. Tumor stages were determined according to the eighth edition of the tumor, node, metastasis (TNM) classification.

All patients with *EGFR* mutations, *ALK*, *ROS1*, and *RET* fusions, and *MET* exon 14 skipping received tyrosine kinase inhibitors as first‐line treatment in our hospital and were therefore excluded from this study. Patients with *BRAF* or *NTRK* alterations were not included in this study, as no such patients were identified during the study period. In Japan, targeted therapies for NSCLC patients with *KRAS* and *HER2* mutations are reimbursed and approved as second‐line treatment. Given that immune checkpoint inhibitors have shown clinical efficacy in this population [22, 23], these patients were included in this study.

Progression of disease was confirmed by investigator assessment according to Response Evaluation Criteria in Solid Tumors version 1.1. Clinical characteristics and pathological data for each patient were extracted by retrospective chart review. Progression‐free survival (PFS) and overall survival (OS) of patients on first‐line treatment were also reviewed.

TTF‐1 expression was evaluated immunohistochemically using formalin‐fixed, paraffin‐embedded (FFPE) tumor specimens, and anti‐TTF‐1 antibody (clone SP141; Roche Diagnostics, Basel, Switzerland) as the primary antibody. Nuclear staining for TTF‐1 in ≥ 10% of tumor cells was considered positive, following widely accepted diagnostic criteria [24].

PD‐L1 expression was evaluated on tumor cells using the 22C3 pharmDx assay (Dako, Agilent Technologies, Santa Clara, CA, USA), categorized based on the tumor proportion score (TPS) into negative (< 1%), intermediate (1%–49%), and high expression (≥ 50%), according to standard clinical practice guidelines [25].

### 2.2 Treatment Exposure and Relative Dose Intensity

Relative dose intensity (RDI) was evaluated during the first four cycles of induction therapy for cytotoxic agents, including platinum compounds, pemetrexed, and nab‐paclitaxel. Actual dose intensity (ADI) was calculated as the total delivered dose divided by the actual treatment duration (weeks), including treatment delays. Standard dose intensity (SDI) was defined according to the planned standard regimen as follows: cisplatin 75 mg/m^2^ on day 1 every 3 weeks, carboplatin area under the curve (AUC) 5–6 on day 1 every 3 weeks, pemetrexed 500 mg/m^2^ on day 1 every 3 weeks, and nab‐paclitaxel 100 mg/m^2^ on day 1, 8, and 15 every 3 weeks. Skipped day 8 and day 15 administrations were recorded as zero dose. RDI was calculated as ADI divided by SDI and expressed as a percentage. ICIs (pembrolizumab and atezolizumab) were not included in the primary RDI analysis; instead, treatment discontinuation and the number of administered cycles were separately evaluated. We defined early discontinuation as treatment discontinuation before completion of four planned induction cycles according to a previous report [26]. Treatment duration was defined as the interval between the treatment initiation date and the last administration date of the regimen, regardless of temporary treatment interruptions. Patients who remained on treatment at the data cutoff date were censored on the date of the last confirmed administration.

### 2.3 Statistical Analysis

The relationship between TTF‐1 expression and patients' clinical characteristics including RDI and median ICI cycle was evaluated using the Mann–Whitney U test for age and Fisher's exact probability test for other variables. PFS was defined as the period from the date of treatment initiation to the date of disease progression or death. OS was defined as the period from the date of treatment initiation to the date of death from any cause. Patients without disease recurrence or death during the observation period were censored at the date of last follow‐up for PFS and OS, respectively (cutoff date: August 31, 2025). PFS, OS and treatment duration were estimated using the Kaplan–Meier method, and patient group differences were compared using the log‐rank test. Hazard ratio (HR) and corresponding 95% confidence intervals (CIs) were calculated from a Cox proportional hazards model. The number of patients with early discontinuation of ICI plus chemotherapy between the Platinum/PEM/Pembro group and the CBDCA/nab‐PTX/Atezo group was compared using the Chi‐square test. Univariate and multivariate Cox proportional hazard model analyses were performed to examine the association between clinical variables and survival. The association between factors considered significant in univariate analysis was confirmed using Spearman's rank correlation coefficient (Rho) analysis; variables with Rho greater than 0.7 were excluded to avoid multicollinearity in the multivariable model. We included variables from the univariate analysis with *p* < 0.20 in the multivariate analysis. We analyzed data using SPSS Statistics (version 31, IBM, Armonk, NY, USA). A *p* < 0.05 was considered statistically significant.

### 2.4 Ethical Considerations

This study was conducted in accordance with the Declaration of Helsinki and was approved by the Hokkaido Cancer Center Institutional Review Board (approval ID 07‐53). The study period extends until December 31, 2026. The need for informed consent was waived owing to the retrospective nature of the study. For patients who were deceased or could not be reached, an opt‐out consent process was implemented.

## 3 Results

### 3.1 Patient Characteristics

**FIGURE 1:**
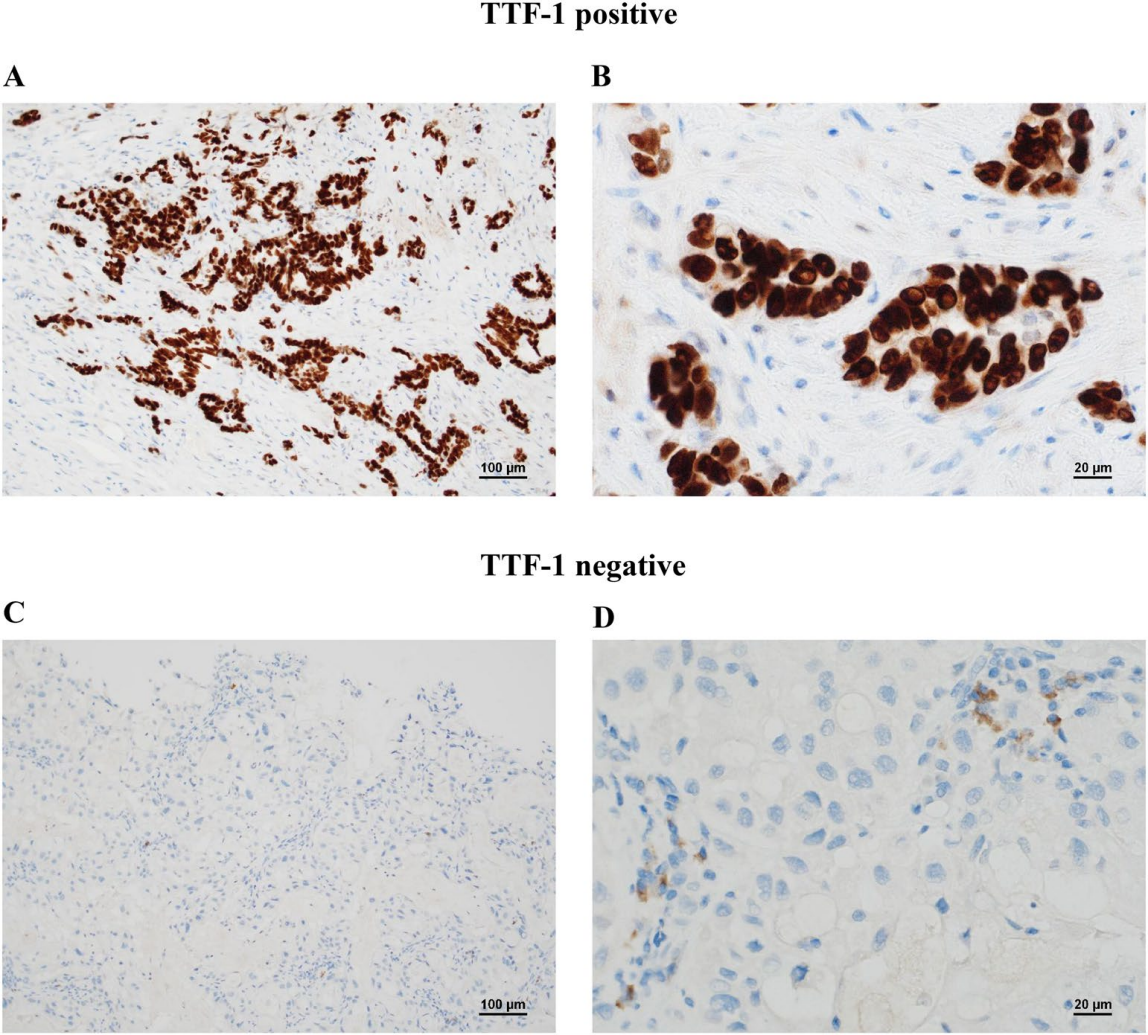
Representative images of immunohistochemically stained tumor sections showing either positive (A and B) or negative (C and D) staining for thyroid transcription factor‐1 (TTF‐1) in cancer cells (original magnification: A and C, 10×; B and D, 40×).

A total of 67 patients with non‐sq NSCLC treated with Platinum/PEM/Pembro or CBDCA/nab‐PTX/Atezo were included in this study. Of these, 44 patients received Platinum/PEM/Pembro, and 23 patients received CBDCA/nab‐PTX/Atezo. Representative images of immunohistochemically stained tumor sections with TTF‐1‐positive and TTF‐1‐negative are shown in Figure 1. The baseline characteristics of the study patients according to the treatment regimens are shown in Table 1. The Platinum/PEM/Pembro group included a higher proportion of younger patients compared to the CBDCA/nab‐PTX/Atezo group (*p* < 0.001). There were significant differences in Eastern Cooperative Oncology Group performance status (ECOG PS) (*p* = 0.025), histological type (*p* = 0.016), and creatinine clearance (*p* = 0.0432) between the two groups; patients in the Platinum/PEM/Pembro group had better PS, a greater frequency of adenocarcinoma, and better renal function. The frequency of *KRAS* mutations was numerically higher in the CBDCA/nab‐PTX/Atezo group, although the difference was not statistically significant (7/44 [15.9%] vs. 7/23 [30.4%], *p* = 0.21). Demographic and clinical characteristics of patients stratified by treatment regimen and according to TTF‐1 expression status are shown in Table 2. Significant differences between the groups were observed in median age (*p* < 0.001), ECOG PS (*p* = 0.0112), histological type (*p* < 0.001), and stage (*p* = 0.0067).

**TABLE 1:** Demographic and clinical characteristics of patients included in this study.

	All patients (*n* = 67)	Platinum + PEM + Pembro (*n* = 44)	CBDCA + nab‐PTX + Atezo (*n* = 23)	*p*
Median age (range)	65.0 (37–83)	65.0 (37–76)	72.0 (58–83)	*p* < 0.001
Sex, *n* (%)				
Male	46 (68.7)	29 (65.9)	17 (73.9)	*p* = 0.502
Female	21 (31.3)	15 (34.1)	6 (26.1)	
ECOG PS, *n* (%)				
0	21 (31.3)	15 (34.1)	6 (26.1)	*p* = 0.025
1	42 (62.7)	29 (65.9)	13 (56.5)	
2	4 (6.0)	0	4 (17.4)	
Smoking status, *n* (%)				
Current	32 (47.8)	22 (50.0)	10 (43.5)	*p* = 0.463
Former	28 (41.8)	19 (43.2)	9 (39.1)	
Never	6 (9.0)	3 (6.8)	3 (13.0)	
Unknown	1 (1.5)	0	1 (4.3)	
Histological type, *n* (%)				
Adenocarcinoma	56 (83.6)	40 (90.9)	16 (69.6)	*p* = 0.016
Favor adenocarcinoma	2 (3.0)	2 (4.5)	0	
NSCLC‐NOS	6 (9.0)	2 (4.5)	4 (17.4)	
LCNEC	1 (1.5)	0	1 (4.3)	
Sarcomatoid carcinomas	2 (3.0)	0	2 (8.7)	
Stage, *n* (%)				
IIIA	1 (1.5)	0	1 (4.3)	*p* = 0.6124
IIIB	2 (3.0)	2 (4.5)	0	
IVA	20 (30.0)	14 (31.8)	6 (26.1)	
IVB	34 (50.7)	22 (50.0)	12 (52.2)	
Recurrence	10 (14.9)	6 (13.6)	4 (17.4)	
PD‐L1 TPS, *n* (%)				
< 1%	27 (40.3)	20 (45.5)	7 (30.4)	*p* = 0.338
1%–49%	23 (34.3)	13 (29.5)	10 (43.5)	
≥ 50%	14 (20.9)	10 (22.7)	4 (17.4)	
Unknown	3 (4.5)	1 (2.3)	2 (8.7)	
Driver mutation, *n* (%)				
Positive	18 (26.9)	11^a^ (25.0)	7^b^ (30.4)	*p* = 0.433
Negative	48 (71.6)	33 (75.0)	15 (65.2)	
Unknown	1 (1.5)	0	1 (4.3)	
Ccr (mL/min), *n* (%)				
Ccr < 45	1 (1.5)	0	1 (4.3)	*p* = 0.0432
45 ≤ Ccr < 60	25 (37.3)	13 (29.5)	12 (52.2)	
60 ≤ Ccr	41 (61.2)	31 (70.5)	10 (43.5)	

*Note:* Platinum/PEM/Pembro, platinum and pemetrexed plus pembrolizumab; CBDCA/nab‐PTX/Atezo, carboplatin and nab‐paclitaxel plus atezolizumab; ECOG PS, Eastern Cooperative Oncology Group Performance Status; PD‐L1, programmed death‐ligand 1; TPS, tumor proportion score; NSCLC‐NOS, non‐small cell lung cancer‐not otherwise specified; LCNEC, large cell neuroendocrine carcinoma; Ccr, creatinine clearance.

^a^ *KRAS* G12C (*n* = 2), *KRAS* G12/13 (*n* = 1), *KRAS* G12D (*n* = 2), *KRAS* G12V (*n* = 2), *HER2* mutation (*n* = 2), *BRAF* non‐V600E mutation (*n* = 2).

^b^ *KRAS* G12C (*n* = 2), *KRAS* G12D (*n* = 5).

**TABLE 2:** Demographic and clinical characteristics of patients stratified by treatment regimen and according to TTF‐1 expression status.

	Platinum + PEM + Pembro TTF‐1 positive (*n* = 35)	Platinum+ PEM+ Pembro TTF‐1 negative (*n* = 9)	CBDCA+ nab‐PTX+ TTF‐1 positive (*n* = 9)	CBDCA+ nab‐PTX+ TTF‐1 negative (*n* = 14)	*p*
Median age (range)	65.0 (37–6)	65.0 (46–74)	77.0 (58–83)	70.5 (59–78)	*p* < 0.001
Sex, *n* (%)					
Male	24 (68.6)	5 (55.6)	6 (66.7)	11 (78.6)	*p* = 0.683
Female	11 (31.4)	4 (44.4)	3 (33.3)	3 (21.4)	
ECOG PS, *n* (%)					
0	15 (42.9)	0	2 (22.2)	4 (28.6)	*p* = 0.0112
1	20 (57.1)	9 (100)	6 (66.7)	7 (50.0)	
2	0	0	1 (11.1)	3 (21.4)	
Smoking status, *n* (%)					
Current	18 (51.4)	4 (44.4)	3 (33.3)	7 (50.0)	*p* = 0.558
Former	15 (42.9)	4 (44.4)	3 (33.3)	6 (42.9)	
Never	2 (5.7)	1 (11.1)	2 (22.2)	1 (7.1)	
Unknown	0	0	1 (11.1)	0	
Histological type, *n* (%)					
Adenocarcinoma	33 (94.3)	7 (77.8)	9 (100)	7 (50.0)	*p* < 0.001
Favor adenocarcinoma	2 (5.7)	0	0	0	
NSCLC‐NOS	0	2 (22.2)	0	4 (28.6)	
LCNEC	0	0	0	1 (7.1)	
Sarcomatoid carcinomas	0	0	0	2 (14.3)	
Stage, *n* (%)					
IIIA	0	0	0 (0)	1 (7.1)	*p* = 0.0067
IIIB	0	2 (22.2)	0	0	
IVA	9 (25.7)	5 (55.6)	1 (11.1)	5 (35.7)	
IVB	20 (57.1)	2 (22.2)	4 (44.4)	8 (57.1)	
Recurrence	6 (17.1)	0	4 (44.4)	0	
PD‐L1 TPS, *n* (%)					
< 1%	18 (51.4)	2 (22.2)	3 (33.3)	4 (28.6)	*p* = 0.307
1%–49%	10 (28.6)	3 (33.3)	4 (44.4)	6 (42.9)	
≥ 50%	7 (20.0)	3 (33.3)	2 (22.2)	2 (14.3)	
Unknown	0	1 (11.1)	0	2 (14.3)	
Driver mutation, *n* (%)					
Positive	10^a^ (28.6)	1^b^ (11.1)	3^c^ (33.3)	4^d^ (28.6)	*p* = 0.565
Negative	25 (71.4)	8 (88.9)	6 (66.7)	9 (64.3)	
Unknown	0	0	0	1 (7.1)	
Ccr (mL/min), *n* (%)					
Ccr < 45	0	0	1 (11.1)	0	*p* = 0.1374
45 ≤ Ccr < 60	11 (31.4)	2 (22.2)	4 (44.4)	8 (57.1)	
60 ≤ Ccr	24 (68.6)	7 (77.8)	4 (44.4)	6 (42.9)	

Abbreviations: CBDCA/nab‐PTX/Atezo, carboplatin and nab‐paclitaxel plus atezolizumab; ECOG PS, Eastern Cooperative Oncology Group Performance Status; NSCLC‐NOS, non‐small cell lung cancer‐not otherwise specified; PD‐L1, programmed death‐ligand 1; Ccr, creatinine clearance; LCNEC, large cell neuroendocrine carcinoma; Platinum/PEM/Pembro, platinum and pemetrexed plus pembrolizumab; TPS, tumor proportion score; TTF‐1, thyroid transcription factor‐1.

^a^ *KRAS* G12C (*n* = 2), *KRAS* G12D (*n* = 2), *KRAS* G12V (*n* = 2), *HER2* mutation (*n* = 2), *BRAF* non‐V600E mutation (*n* = 2).

^b^ *KRAS* G12/13 (*n* = 1).

^c^ *KRAS* G12C (*n* = 1), *KRAS* G12D (*n* = 2).

^d^ *KRAS* G12C (*n* = 1), *KRAS* G12D (*n* = 3).

### 3.2 PFS and OS in the Overall Population

**FIGURE 2:**
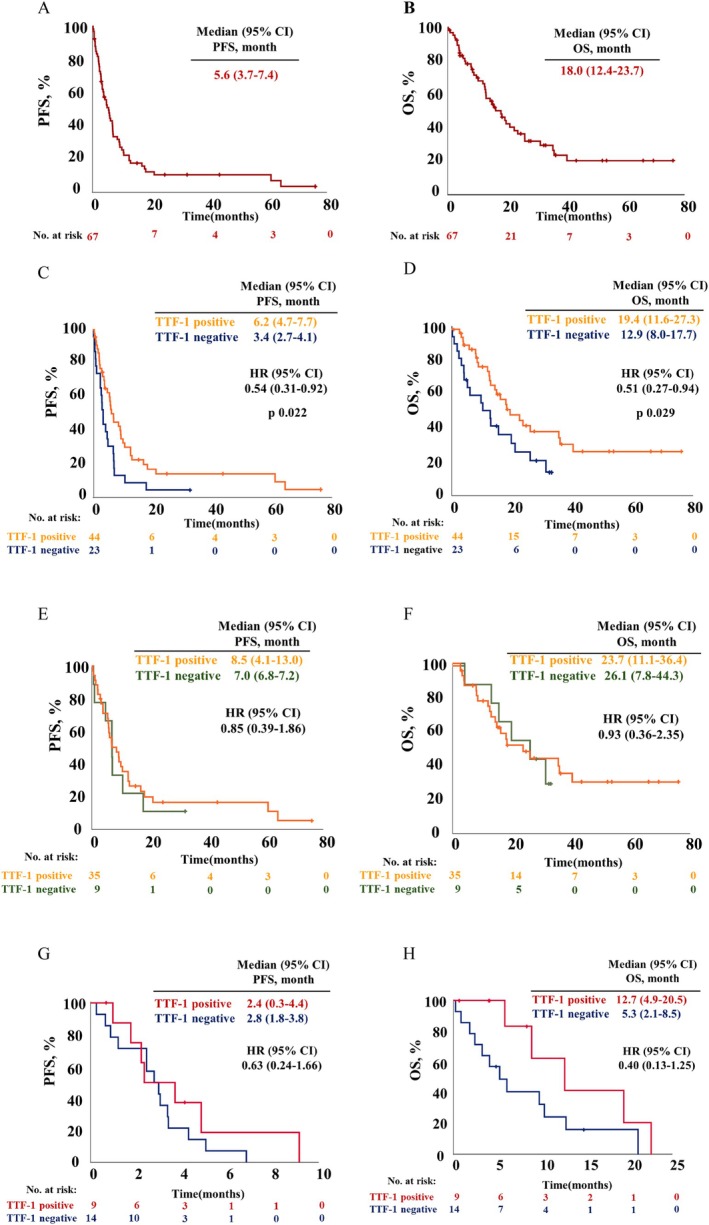
Kaplan–Meier curves of PFS (A) and OS (B) in the overall population. Kaplan–Meier curves according to TTF‐1 expression status: PFS (C) and OS (D) in the overall population; PFS (E) and OS (F) in patients treated with Platinum/PEM/Pembro; PFS (G) and OS (H) in the patients treated with CBDCA/nab‐PTX/Atezo. PFS, progression‐free survival; OS, overall survival; No., number; TTF‐1, thyroid transcription factor‐1; CI, confidence interval; HR, hazard ratio; Platinum/PEM/Pembro, Platinum and pemetrexed plus pembrolizumab; CBDCA/nab‐PTX/Atezo, carboplatin and nab‐paclitaxel plus atezolizumab.

Kaplan–Meier curves for PFS and OS in the overall population (*n* = 67) are shown in Figure 2A,B, respectively. With a median follow‐up of 13.0 months (0.3–76.2 months), the median PFS was 5.6 months (95% CI, 3.7–7.4 months), and the median OS was 18.0 months (95% CI, 12.4–23.7 months).

In the analysis stratified by TTF‐1 expression, TTF‐1‐positive patients had a longer PFS than TTF‐1‐negative patients (median, 6.2 vs. 3.4 months; HR, 0.54; 95% CI, 0.31–0.92, *p* = 0.022) (Figure 2C). Similarly, TTF‐1‐positive patients had a longer OS than TTF‐1‐negative patients (median, 19.4 vs. 12.9 months; HR, 0.51; 95% CI, 0.27–0.94, *p* = 0.029) (Figure 2D).

### 3.3 PFS And OS According to Chemoimmunotherapy Regimen

Kaplan–Meier curves for PFS and OS in the Platinum/PEM/Pembro group and the CBDCA/nab‐PTX/Atezo group are shown in Figure 2E–H.

In the analysis of the Platinum/PEM/Pembro group, there were no significant differences in PFS or OS between TTF‐1‐positive patients and TTF‐1‐negative patients (median PFS: 8.5 vs. 7.0 months; HR, 0.85; 95% CI, 0.39–1.86, and median OS: 23.7 vs. 26.1 months; HR, 0.93; 95% CI, 0.36–2.35, respectively). Similarly, in the analysis of the CBDCA/nab‐PTX/Atezo group, there was no significant difference in PFS between TTF‐1‐positive patients and TTF‐1‐negative patients (2.4 vs. 2.8 months; HR, 0.63; 95% CI, 0.24–1.66). There was also no significant difference in OS, but OS was numerically shorter in TTF‐1‐negative patients than in TTF‐1‐positive patients (12.7 vs. 5.3 months; HR, 0.40; 95% CI, 0.13–1.25).

### 3.4 Swimmer's Plots of the Platinum/PEM/Pembro Group and the CBDCA/Nab‐PTX/Atezo Group According to TTF‐1 Expression

**FIGURE 3:**
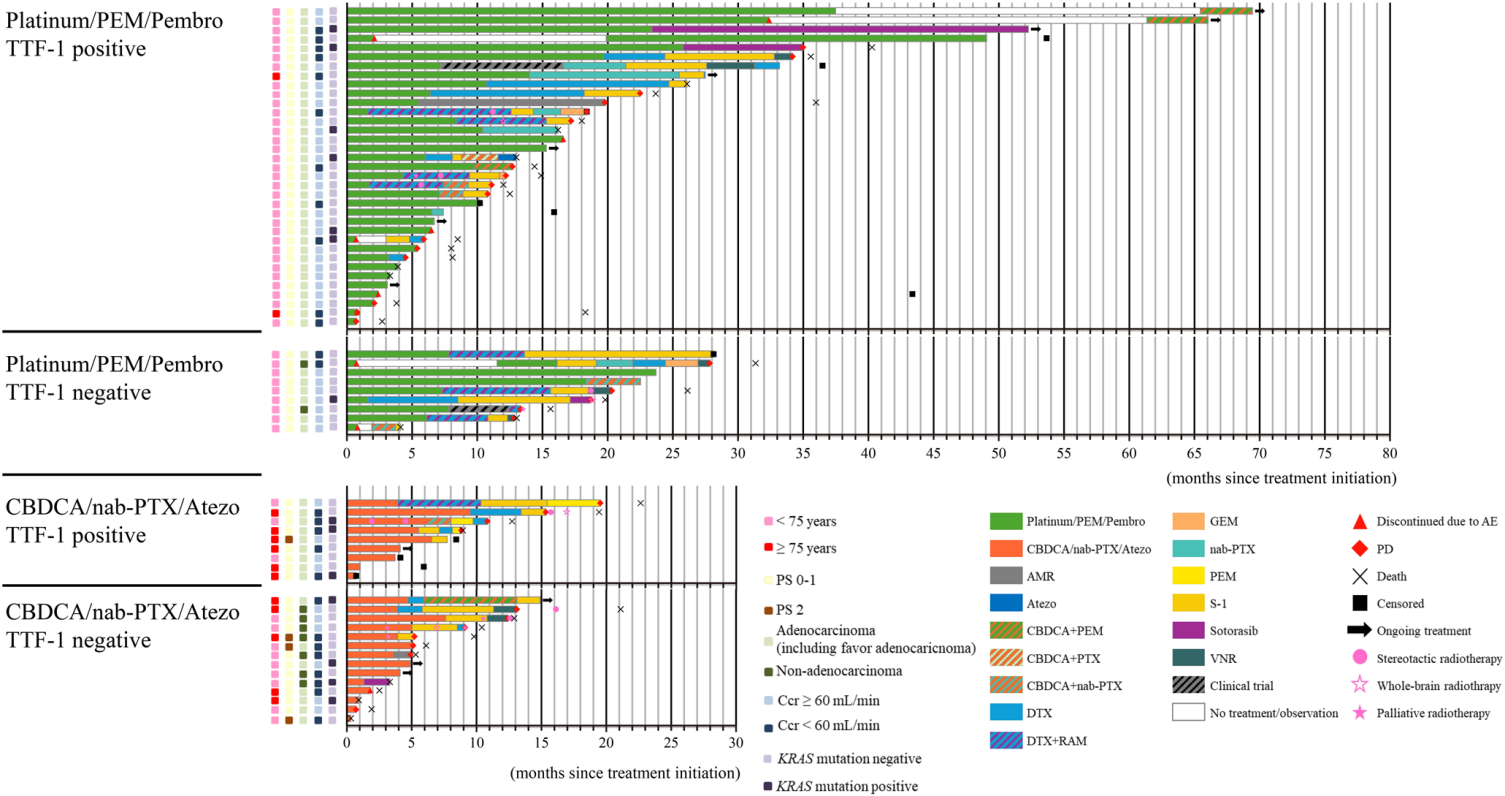
Swimmer's plots of the Platinum/PEM/Pembro group and the CBDCA/nab‐PTX/Atezo group according to TTF‐1 expression. TTF‐1, thyroid transcription factor‐1; PEM, pemetrexed; Pembro, pembrolizumab; CBDCA, carboplatin; nab‐PTX, nab‐paclitaxel; Atezo, atezolizumab; PS, performance status; Ccr, creatinine clearance; AMR, amurubicin; PTX, paclitaxel; DTX, docetaxel; RAM, ramucirumab; GEM, gemcitabine; S‐1, tegafur/gimeracil/oteracil potassium; VNR, vinorelbine; AE, adverse event; PD, progressive disease.

The swimmer plots for the 67 patients analyzed in this study are shown in Figure 3. The treatment duration in the Platinum/PEM/Pembro group was relatively long not only in the first‐line setting but also in subsequent therapy. Furthermore, some patients who had discontinued first‐line treatment because of adverse events achieved a response after treatment reinitiation.

### 3.5 Treatment Exposure and Relative Dose Intensity According to Chemoimmunotherapy Regimen

Table 3 summarizes treatment intensity and duration in the Platinum/PEM/Pembro and CBDCA/nab‐PTX/Atezo groups. The RDI of platinum and concomitant chemotherapy was 100% vs. 85.7% (*p* = 0.072) and 92.3% vs. 58.8% (*p* < 0.001), respectively. The median number of ICI cycles was 6 vs. 3 (*p* = 0.004), respectively. Early discontinuation of ICI plus chemotherapy occurred in 14 patients (31.8%) vs. 15 patients (65.2%) (*p* = 0.009), respectively. The median treatment duration was 5.49 vs. 2.14 months (*p* = 0.001), respectively.

**TABLE 3:** Treatment intensity and duration of each regimen.

	Platinum/PEM/Pembrogroup	CBDCA/nab‐PTX/Atezogroup	*p*
Median platinum RDI (range)	100% (22.2–100)	85.7% (52.3–100)	0.072
Median RDI of concomitant chemotherapy (range)	92.3% (7.1–100)^a^	58.8% (33.3–100)^b^	< 0.001
Median ICI cycles (range)	6 (1–35)	3 (1–9)	0.004
Early discontinuation of ICI + chemotherapy (< 4 cycles), *n* (%)	14 (31.8)	15 (65.2)	0.009
Median treatment duration (95% CI)	5.49 months (4.30–6.61)	2.14 months (0.10–5.00)	0.001

Abbreviations: CBDCA/nab‐PTX/Atezo, carboplatin and nab‐paclitaxel plus atezolizumab; CI, confidence interval; ICI, immune checkpoint inhibitor; Platinum/PEM/Pembro, platinum and pemetrexed plus pembrolizumab; RDI, relative dose intensity.

^a^ Pemetrexed.

^b^ nab‐paclitaxel.

### 3.6 Univariate and Multivariate Analyses of Factors Associated With PFS and OS in All Patients

Spearman's rank correlation coefficient (Rho) indicated a moderate correlation between TTF‐1 positivity and adenocarcinoma histology (Rho = 0.545), while no significant correlations were observed among the other variables. Univariate and multivariate Cox proportional hazards models were constructed to evaluate the prognostic impact of TTF‐1 expression, age, ECOG PS, PD‐L1 expression, histological type, smoking history, renal function, and *KRAS* mutation status (Table 4). In univariate analysis, TTF‐1 expression and PS were significantly associated with PFS, while TTF‐1 expression, PS, and histological type were significantly associated with OS. However, no independent prognostic factors were identified in multivariate analysis.

**TABLE 4:** Univariate and multivariate analyses of factors associated with PFS and OS in all patients.

PFS	Univariate		Multivariate	
Covariates	HR (95% CI)	*p*	HR (95% CI)	*p*
TTF‐1 (positive vs. negative)	0.54 (0.31–0.92)	0.022	0.59 (0.31–1.15)	0.12
Age (< 75 vs. ≥ 75 years)	0.65 (0.33–1.26)	0.20		
ECOG PS (< 2 vs. ≥ 2)	0.29 (0.10–0.83)	0.022	0.44 (0.15–1.30)	0.14
PD‐L1 (≥ 50% vs. < 50%)	0.57 (0.29–1.12)	0.11	0.54 (0.27–1.06)	0.074
Histology (adeno vs. non‐adeno)	0.55 (0.27–1.14)	0.11	0.76 (0.32–1.80)	0.53
Smoking history (yes vs. no)	0.78 (0.33–1.84)	0.57		
Renal function (Ccr < 60 vs. ≥ 60)	0.86 (0.50–1.45)	0.57		
*KRAS* mutation (yes vs. no)	0.82 (0.43–1.56)	0.55		

OS	Univariate		Multivariate	
Covariates	HR (95% CI)	*p*	HR (95% CI)	*p*
TTF‐1 (positive vs. negative)	0.51 (0.27–0.94)	0.032	0.66 (0.30–1.43)	0.29
Age (< 75 vs. ≥ 75 years)	0.65 (0.30–1.42)	0.28		
ECOG PS (< 2 vs. ≥ 2)	0.26 (0.08–0.91)	0.035	0.36 (0.10–1.26)	0.11
PD‐L1 (≥ 50% vs. < 50%)	0.72 (0.34–1.51)	0.38		
Histology (adeno vs. non‐adeno)	0.43 (0.20–0.90)	0.026	0.64 (0.25–1.64)	0.35
Smoking history (yes vs. no)	0.83 (0.29–2.32)	0.72		
Renal function (Ccr < 60 vs. ≥ 60)	0.88 (0.48–1.63)	0.69		
*KRAS* mutation (yes vs. no)	0.91 (0.42–1.98)	0.82		

*Note:* Cox proportional hazard model analysis was used to obtain the *p* values.

Abbreviations: adeno, adenocarcinoma (including favor adenocarcinoma); Ccr, creatinine clearance; CI, confidence interval; ECOG PS, Eastern Cooperative Oncology Group Performance Status; HR, hazard ratio; non‐adeno, non‐adenocarcinoma; OS, overall survival; PD‐L1, programmed death‐ligand 1; PFS, progression‐free survival; TTF‐1, thyroid transcription factor‐1.

### 3.7 Safety

Table 5 summarizes treatment‐emergent adverse events in the Platinum/PEM/Pembro group and the CBDCA/nab‐PTX/Atezo group. Grade 3 or higher anemia (*p* = 0.042), and thrombocytopenia (*p* = 0.037) were more frequently observed in the CBDCA/nab‐PTX/Atezo group. Immune‐related adverse events occurred more frequently in the Platinum/PEM/Pembro group than in the CBDCA/nab‐PTX/Atezo group (50.0% vs. 17.4%, *p* = 0.0164) (Table 6). Although no statistically significant difference was observed in the incidence of pneumonitis (*p* = 0.149), it was numerically higher in the Platinum/PEM/Pembro group (18.2% vs. 4.3%). A summary of treatment interruptions, dose reductions, and discontinuations is shown in Table 7. The first two events were largely attributable to the aforementioned myelosuppression. Dose reductions were less frequent in the Platinum/PEM/Pembro group than in the CBDCA/nab‐PTX/Atezo group (2.3% [1/44] vs. 26.1% [6/23], *p* = 0.00539).

**TABLE 5:** Summary of treatment‐emergent adverse events.

Event, *n* (%)	Platinum/PEM/Pembro		CBDCA/nab‐PTX/Atezo	
	Any grade	Grade 3–5	Any grade	Grade 3–5
Leukopenia	13 (29.5)	1 (2.3)	12 (52.2)	2 (8.7)
Neutropenia	17 (38.6)	10 (22.7)	16 (69.6)	11 (47.8)
Febrile neutropenia	1 (2.3)	1 (2.3)	1 (4.3)	1 (4.3)
Anemia	20 (45.5)	2 (4.5)	20 (87.0)	5 (21.8)
Thrombocytopenia	11 (25.0)	0	14 (60.9)	3 (13.0)
Increased aspartate aminotransferase	21 (47.7)	2 (4.5)	11 (47.8)	1 (4.3)
Increased alanine aminotransferase	21 (47.7)	5 (11.3)	9 (39.1)	1 (4.3)
Increased creatinine	2 (4.5)	0	3 (13.0)	0
Hyponatremia	1 (2.3)	0	3 (13.0)	0
Hypokalemia	1 (2.3)	1 (2.3)	1 (4.3)	1 (4.3)
Hyperkalemia	4 (9.1)	0	0	0
Nausea	12 (27.3)	2 (4.5)	1 (4.3)	0
Constipation	11 (25.0)	0	4 (17.4)	1 (4.3)
Diarrhea	5 (11.4)	1 (2.3)	1 (4.3)	0
Fatigue	6 (13.6)	0	3 (13.0)	0
Decreased appetite	9 (20.5)	2 (4.5)	2 (8.7)	0
Dysgeusia	2 (4.5)	0	0	0
Myalgia	0	0	1 (4.3)	1 (4.3)
Arthralgia	2 (4.5)	0	1 (4.3)	1 (4.3)
Peripheral neuropathy	2 (4.5)	0	1 (4.3)	0
COVID‐19	1 (2.3)	1 (2.3)	0	0
Bacterial pneumonia	0	0	2 (8.7)	2 (8.7)
Aortic dissection	0	0	1 (4.3)	1 (4.3)
Gastric ulcer	1 (2.3)	0	0	0
Lower gastrointestinal perforation	0	0	1 (4.3)	1 (4.3)

Abbreviations: CBDCA/nab‐PTX/Atezo, carboplatin and nab‐paclitaxel plus atezolizumab; Platinum/PEM/Pembro, platinum and pemetrexed plus pembrolizumab.

**TABLE 6:** Summary of immune‐related adverse events.

Event, *n* (%)	Platinum/PEM/Pembro		CBDCA/nab‐PTX/Atezo	
	Any grade	Grade 3–5	Any grade	Grade 3–5
Total	22 (50.0)	4 (9.1)	4 (17.4)	2 (8.7)
Pneumonitis	8 (18.2)	2 (4.5)	1 (4.3)	1 (4.3)
Rash	11 (25.0)	1 (2.3)	2 (8.7)	0
Colitis	2 (4.5)	1 (2.3)	1 (4.3)	1 (4.3)
Hyperthyroidism	1 (2.3)	0	0	0
Hypothyroidism	2 (4.5)	0	1 (4.3)	0
Adrenal insufficiency	2 (4.5)	0	0	0
Encephalitis	1 (2.3)	0	0	0
Myasthenia gravis	2 (4.5)	0	0	0
Hypohidrosis	1 (2.3)	0	0	0
Infusion‐related reaction	1 (2.3)	0	0	0

Abbreviations: CBDCA/nab‐PTX/Atezo, carboplatin and nab‐paclitaxel plus atezolizumab; Platinum/PEM/Pembro, platinum and pemetrexed plus pembrolizumab.

**TABLE 7:** Summary of treatment interruptions, dose reductions, and discontinuations.

Event, *n* (%)	Platinum/PEM/Pembro	CBDCA/nab‐PTX/Atezo
Interruptions	3 (6.8)	4 (17.4)
Dose reductions	1 (2.3)	6 (26.1)
Discontinuations	7 (15.9)	1 (4.3)

Abbreviations: CBDCA/nab‐PTX/Atezo, carboplatin and nab‐paclitaxel plus atezolizumab; Platinum/PEM/Pembro, platinum and pemetrexed plus pembrolizumab.

## 4 Discussion

This is one of the largest studies examining the outcomes of Platinum/PEM/Pembro and CBDCA/nab‐PTX/Atezo in patients with patients according to TTF‐1 expression.

Previous reports have described multiple studies evaluating efficacy according to TTF‐1 expression in NSCLC patients treated with immune checkpoint inhibitor monotherapy or chemoimmunotherapy. A meta‐analysis of studies on chemoimmunotherapy demonstrated that TTF‐1‐negative patients had shorter PFS and OS than TTF‐1‐positive patients, indicating a poorer prognosis [20]. However, the study did not demonstrate the comparative effect of different regimens. Our overall cohort showed similar results and clearly demonstrated regimen‐specific clinical outcomes. TTF‐1 expression has long been associated with favorable outcomes and enhanced sensitivity to pemetrexed‐based chemotherapy [16, 17, 18, 19], whereas non‐PEM‐based regimens including taxanes may confer better outcomes in TTF‐1‐negative patients [16].

However, since the introduction of chemoimmunotherapy, the optimal regimen for TTF‐1‐negative cases has not been fully investigated. Nishioka et al. [27] compared PEM‐based chemoimmunotherapy with non‐PEM‐based chemoimmunotherapy in PD‐L1‐high non‐sq NSCLC patients and reported a significantly better PFS with PEM‐based chemoimmunotherapy regardless of TTF‐1 expression, while OS showed only a non‐significant trend toward improvement. They also reported no difference in PFS and OS between PEM‐based and non‐PEM‐based chemoimmunotherapy in TTF‐1‐negative NSCLC patients with PD‐L1 expression of 1%–49% [28]. In a prospective study of TTF‐1‐negative NSCLC, Shiraishi et al. [21] reported a median PFS of 4.9 months (80% CI, 4.3–5.9), but the primary endpoint was not met as the lower CI bound did not exceed the prespecified 4.5‐month threshold. In addition, real‐world evidence on the efficacy of taxane‐based chemoimmunotherapy according to TTF‐1 expression status remains limited. Based on these findings, the optimal PEM‐based or taxane‐based chemoimmunotherapy regimen stratified by TTF‐1 expression remains unclear, indicating the need for further studies.

In this cohort, among TTF‐1‐negative non‐sq NSCLC patients, Platinum/PEM/Pembro showed PFS and OS comparable to those observed in TTF‐1‐positive patients. In this group, the median PFS and OS were > 7.0 months and > 23 months, respectively, comparable to those reported in major chemoimmunotherapy trials, including KEYNOTE‐189 [5], IMpower130 [6], IMpower150 [29], CheckMate 9LA [30], and POSEIDON [31]. Although the retrospective nature of this study should be acknowledged as a limitation, the present results indicate that Platinum/PEM/Pembro may be a viable chemoimmunotherapy option for TTF‐1‐negative cases.

However, patient characteristics differed between the Platinum/PEM/Pembro group and the CBDCA/nab‐PTX/Atezo group. The differences in baseline characteristics may be explained by the preference of the attending physician, considering several factors; (i) in patients aged ≥ 75 years, the subgroup analysis of KEYNOTE‐189 trial did not demonstrate a clear superiority of Platinum/PEM/Pembro over Platinum/PEM alone in terms of PFS or OS [32]. (ii) a pooled analysis of the IMpower130 and IMpower132 trials showed that atezolizumab plus chemotherapy was associated with significant improvements in both PFS and OS compared with chemotherapy alone in elderly patients [33]. (iii) taxane‐based chemotherapy has been suggested to be more effective in patients with TTF‐1‐negative tumors [16] or those with NSCLC not otherwise specified histology [34]. As a result, the CBDCA/nab‐PTX/Atezo group included a higher proportion of elderly patients and those with non‐adenocarcinoma histology. In the CBDCA/nab‐PTX/Atezo group, the swimmer's plot showed that shorter treatment durations were more common among elderly patients (≥ 75 years), those with PS 2, and those with non‐adenocarcinoma histology. Previous studies have reported limited treatment efficacy in these populations [35, 36, 37, 38, 39]. These findings may partly reflect the higher proportion of patients with these characteristics in the CBDCA/nab‐PTX/Atezo group, which had significantly lower treatment intensity and shorter treatment duration than the Platinum/PEM/Pembro group.

In the overall population, TTF‐1 positivity was associated with a significantly longer PFS compared with TTF‐1 negativity, consistent with the findings reported by Katayama et al. [40], whose study included both Platinum/PEM/Pembro and CBDCA/nab‐PTX/Atezo regimens. Mori et al. [41] reported that in patients treated with Platinum/PEM/Pembro alone, no significant difference in PFS was observed between TTF‐1‐positive and TTF‐1‐negative cases, which was also consistent with our findings. In contrast, Ibusuki et al. [42] demonstrated that the PFS of patients treated with Platinum/PEM/Pembro alone was significantly longer in TTF‐1‐positive cases than in TTF‐1‐negative cases. These discrepant results may suggest differences in patient baseline characteristics across these studies.

Indeed, when the analysis was limited to pemetrexed‐containing regimens, all TTF‐1‐negative cases in our study had a PS of 1, whereas the proportions of patients with PS of 1 were 50% and 64% in the reports by Katayama et al. and Ibusuki et al., respectively. In the report by Mori et al., PS was not described. The proportion of PD‐L1‐negative cases among TTF‐1‐positive patients was 51.4% in our study, whereas the corresponding proportions were 16%, 30.7%, and 21.3% in the reports by Katayama et al., Mori et al., and Ibusuki et al., respectively, suggesting notable differences in PD‐L1 status distribution among studies. Furthermore, male patients accounted for 55.6% of TTF‐1‐negative cases in our study, compared with 95.0%, 72.3%, and 71.2% in the reports. *KRAS* mutations were detected in 17% of patients in our cohort, whereas the frequency was 5.3% in the report by Katayama et al.

There were no substantial differences in median age or smoking history across the studies. Regarding histology, non‐adenocarcinoma histology accounted for 22.2% of TTF‐1‐negative cases in our study, whereas the study by Katayama et al. included only adenocarcinoma cases, the study by Ibusuki et al. included 23.4% of non‐adenocarcinoma cases among TTF‐1‐negative patients, and the study by Mori et al. included only non‐sq NSCLC cases, indicating considerable differences in histological background across the studies. Ours was also the only study that evaluated renal function. Furthermore, although Mori et al. assessed treatment discontinuation, none of the previous studies investigated the actual treatment duration or RDI.

Given these variations in clinical characteristics across studies, we investigated the clinical factors independently associated with PFS and OS. In univariate analysis, TTF‐1 expression and PS were significantly associated with PFS, while TTF‐1 expression, PS, and histological type were significantly associated with OS. TTF‐1 positivity was moderately correlated with adenocarcinoma histology, indicating collinearity between these two variables; nevertheless, as they are not interchangeable, both were included in the multivariate model. However, no independent prognostic factors were identified in multivariate analysis, possibly reflecting the collinearity between TTF‐1 expression and histological type, which may have attenuated the individual contribution of each variable, as well as the limited sample size. Further studies with large cohorts are warranted to confirm these findings.

Among TTF‐1‐negative cases, the Platinum/PEM/Pembro group included one *KRAS* mutation case (PD‐L1 TPS was 25%–49%), whereas the CBDCA/nab‐PTX/Atezo group had four such cases (PD‐L1 TPS values were < 1% in two patients, one patient had 1%, and one patient had 1%–24%), representing a higher proportion. In *KRAS* mutation‐positive cases, *KEAP1*/*STK11* co‐mutations may occur [43, 44]. Loss‐of‐function mutations in *STK11* or *KEAP1* create metabolically suppressed, poorly inflamed microenvironments and lower PD‐L1 expression [45], and confer resistance to anti‐PD‐1 antibodies such as pembrolizumab monotherapy and chemoimmunotherapy in non‐sq NSCLC [45, 46, 47, 48, 49]. Additionally, TTF‐1‐negative lung adenocarcinoma has been reported to harbor *KEAP1* mutations more frequently than TTF‐1‐positive cases [50, 51]. Preclinical models have also shown that TTF‐1 negativity is associated with *STK11* loss [52]. In this study, *KEAP1/STK11* gene alterations were not assessed, and *KRAS* mutation status was not identified as an independent prognostic factor for PFS or OS in multivariate analysis. Nevertheless, a disproportionate distribution of these genomic factors in the CBDCA/nab‐PTX/Atezo group may have contributed to the poorer outcomes observed among TTF‐1‐negative cases.

The Platinum/PEM/Pembro group demonstrated an incidence of irAE pneumonitis of 18.2%. In the KEYNOTE‐189 trial, the incidence of irAE pneumonitis was 4.9% [53]. However, real‐world data from Japanese patients reported an 18% incidence [54], consistent with our findings. The higher incidence of irAE in the Platinum/PEM/Pembro group compared with CBDCA/nab‐PTX/Atezo may be attributed to PD‐1 inhibitors generally causing more irAE than PD‐L1 inhibitors [55], as well as the longer treatment duration in the Platinum/PEM/Pembro group, which may have increased the opportunity for irAE to occur. Alternatively, the higher incidence of irAEs may also have contributed to the longer PFS and OS [56, 57, 58] observed in this group.

This study has several limitations. First, it was a single‐center retrospective study with a relatively small sample size. After classifying patients by treatment regimen, the ability to detect significant differences in efficacy was reduced. Selection bias in regimen choice may have been present, influenced by patient age, PS, and histologic type as described previously. Adjustment for confounding factors using methods such as propensity score matching was difficult because of the small sample size. Therefore, a direct comparison between the Platinum/PEM/Pembro and CBDCA/nab‐PTX/Atezo groups was considered inappropriate and was not performed in this study. Confirmation by accumulation of cases across multiple centers using score matching or prospective trials is therefore necessary. An ongoing phase II trial (AIO‐TRK‐0122, ANTELOPE trial) comparing Platinum/PEM/Pembro and CBDCA/nab‐PTX/Atezo in TTF‐1‐negative advanced or postoperative recurrent non‐sq NSCLC may provide further insights into the selection of systemic therapy for this population [59]. In addition, a phase II trial (WJOG17223L, TURNING) evaluating CBDCA, nab‐PTX, tremelimumab, and durvalumab for patients with TTF‐1‐negative NSCLC is currently underway [60]. Second, the study did not evaluate mutation status, including *KEAP1/STK11* as described above. This limitation is attributable to restrictions imposed by the Japanese health insurance system, which currently does not allow upfront comprehensive genomic profiling, and this remains an issue to be addressed in the future. Third, given the retrospective design of this study, differences in imaging assessment schedules among individual physicians may have contributed to the observed differences in PFS. Fourth, the appropriateness of the safety assessment requires careful interpretation.

In conclusion, TTF‐1‐negative patients had shorter PFS and OS compared with TTF‐1‐positive patients in the chemoimmunotherapy setting. In this study, the CBDCA/nab‐PTX/Atezo group was characterized by a higher proportion of poor PS and non‐adenocarcinoma histology. Effective treatment strategies for this population remain a significant unmet medical need, highlighting the necessity for future therapeutic development. Among TTF‐1‐negative patients, Platinum/PEM/Pembro demonstrated comparable efficacy to that observed in TTF‐1‐positive patients and may represent a viable treatment option, particularly for those with good PS and adenocarcinoma histology. However, careful attention should be paid to adverse events, including irAEs.

## Author Contributions

**Ryohei Kamada:** conceptualization, data curation, formal analysis, investigation, visualization, writing – original draft, writing – review and editing. **Hiroshi Yokouchi:** conceptualization, data curation, formal analysis, investigation, project administration, visualization, writing – original draft, writing – review and editing. **Shohei Mizobuchi:** investigation, resources, writing – review and editing. **Hidenori Mizugaki:** investigation, resources, writing – review and editing. **Noriyuki Yamada:** investigation, resources, writing – review and editing. **Yasushi Mizukami:** investigation, resources, writing – review and editing. **Yoshihiro Matsuno:** investigation, formal analysis, visualization, writing – review and editing. **Hajime Asahina:** investigation, resources, writing – review and editing. **Hirofumi Adachi:** investigation, resources, writing – review and editing. **Satoshi Oizumi:** supervision, validation, writing – review and editing. **Ken Kuwahara:** investigation, formal analysis, visualization, writing – review and editing.

## Funding

This work was supported by research funding from the Department of Respiratory Medicine, NHO Hokkaido Cancer Center. The study sponsors had no role in the study design, data collection, data interpretation, writing of the report, or the decision to submit the paper for publication. Assistance with English proofreading was funded by the Department of Respiratory Medicine, NHO Hokkaido Cancer Center.

## Ethics Statement

The study was conducted in accordance with the Declaration of Helsinki and was approved by the Hokkaido Cancer Center Institutional Review Board (approval ID 07 53).

## Consent

The requirement for informed consent was waived because of the retrospective nature of the study. For patients who were deceased or could not be contacted, an opt‐out consent process was used.

## Conflicts of Interest

Hiroshi Yokouchi received grants from AbbVie, Amgen, AstraZeneca, Bristol‐Myers Squibb, Chugai Pharmaceutical, Daiichi‐Sankyo, MSD, Sanofi, and Takeda Pharmaceuticals, and honoraria for lectures from AstraZeneca and Chugai Pharmaceutical. Hidenori Mizugaki received research funding from AstraZeneca and Boehringer Ingelheim, and honoraria from MSD, Bristol‐Myers Squibb, AstraZeneca, Chugai, and Daiichi‐Sankyo. Hajime Asahina received research funding from AstraZeneca and honoraria from MSD, Ono Pharmaceutical, Bristol‐Myers Squibb, AstraZeneca, Chugai, Eli Lilly, Kyowa Hakko Kirin, and Daiichi‐Sankyo. Satoshi Oizumi received grants from AbbVie, Amgen, AstraZeneca, Bristol‐Myers Squibb, Chugai Pharmaceutical, Daiichi‐Sankyo, MSD, Sanofi, and Takeda Pharmaceuticals, and honoraria for lectures from AstraZeneca, Chugai Pharmaceutical, and MSD. The other authors declare no conflicts of interest.

## Data Availability

The data that support the findings of this study are available from the corresponding author upon reasonable request.

## References

[1] H. Sung , J. Ferlay , R. L. Siegel , et al., “Global Cancer Statistics 2020: GLOBOCAN Estimates of Incidence and Mortality Worldwide for 36 Cancers in 185 Countries,” CA: a Cancer Journal for Clinicians 71 (2021): 209–249. 33538338 10.3322/caac.21660

[2] H. Borghaei , L. Paz‐Ares , L. Horn , et al., “Nivolumab Versus Docetaxel in Advanced Nonsquamous Non‐Small‐Cell Lung Cancer,” New England Journal of Medicine 373 (2015): 1627–1639. 26412456 10.1056/NEJMoa1507643 PMC5705936

[3] M. Reck , D. Rodríguez‐Abreu , A. G. Robinson , et al., “Pembrolizumab Versus Chemotherapy for PD‐L1‐Positive Non‐Small‐Cell Lung Cancer,” New England Journal of Medicine 375 (2016): 1823–1833. 27718847 10.1056/NEJMoa1606774

[4] A. Rittmeyer , F. Barlesi , D. Waterkamp , et al., “Atezolizumab Versus Docetaxel in Patients With Previously Treated Non‐Small‐Cell Lung Cancer (OAK): A Phase 3, Open‐Label, Multicentre Randomised Controlled Trial,” Lancet 389 (2017): 255–265. 27979383 10.1016/S0140-6736(16)32517-X PMC6886121

[5] L. Gandhi , D. Rodríguez‐Abreu , S. Gadgeel , et al., “Pembrolizumab Plus Chemotherapy in Metastatic Non‐Small‐Cell Lung Cancer,” New England Journal of Medicine 378 (2018): 2078–2092. 29658856 10.1056/NEJMoa1801005

[6] H. West , M. McCleod , M. Hussein , et al., “Atezolizumab in Combination With Carboplatin Plus Nab‐Paclitaxel Chemotherapy Compared With Chemotherapy Alone as First‐Line Treatment for Metastatic Non‐Squamous Non‐Small‐Cell Lung Cancer (IMpower130): A Multicentre, Randomised, Open‐Label, Phase 3 Trial,” Lancet Oncology 20 (2019): 924–937. 31122901 10.1016/S1470-2045(19)30167-6

[7] M. D. Bruno , R. J. Bohinski , K. M. Huelsman , J. A. Whitsett , and T. R. Korfhagen , “Lung Cell‐Specific Expression of the Murine Surfactant Protein A (SP‐A) Gene Is Mediated by Interactions Between the SP‐A Promoter and Thyroid Transcription Factor‐1,” Journal of Biological Chemistry 270 (1995): 6531–6536. 7896788 10.1074/jbc.270.12.6531

[8] S. E. Kelly , C. J. Bachurski , M. S. Burhans , and S. W. Glasser , “Transcription of the Lung‐Specific Surfactant Protein C Gene Is Mediated by Thyroid Transcription Factor 1,” Journal of Biological Chemistry 271 (1996): 6881–6888. 8636114 10.1074/jbc.271.12.6881

[9] L. Zhang , J. A. Whitsett , and B. R. Stripp , “Regulation of Clara Cell Secretory Protein Gene Transcription by Thyroid Transcription Factor‐1,” Biochimica et Biophysica Acta 1350 (1997): 359–367. 9061032 10.1016/s0167-4781(96)00180-7

[10] Y. Yatabe , T. Mitsudomi , and T. Takahashi , “TTF‐1 Expression in Pulmonary Adenocarcinomas,” American Journal of Surgical Pathology 26 (2002): 767–773. 12023581 10.1097/00000478-200206000-00010

[11] A. S. Abutaily , B. J. Addis , and W. R. Roche , “Immunohistochemistry in the Distinction Between Malignant Mesothelioma and Pulmonary Adenocarcinoma: A Critical Evaluation of New Antibodies,” Journal of Clinical Pathology 55 (2002): 662–668. 12194995 10.1136/jcp.55.9.662 PMC1769743

[12] K. Bakir , N. E. Koçer , H. Deniz , and M. E. Güldür , “TTF‐1 and Surfactant‐B as Co‐Adjuvants in the Diagnosis of Lung Adenocarcinoma and Pleural Mesothelioma,” Annals of Diagnostic Pathology 8 (2004): 337–341. 15614737 10.1053/j.anndiagpath.2004.08.003

[13] G. Pelosi , A. Scarpa , F. Forest , and A. Sonzogni , “The Impact of Immunohistochemistry on the Classification of Lung Tumors,” Expert Review of Respiratory Medicine 10 (2016): 1105–1121. 27617475 10.1080/17476348.2017.1235975

[14] M. M. Winslow , T. L. Dayton , R. G. W. Verhaak , et al., “Suppression of Lung Adenocarcinoma Progression by Nkx2‐1,” Nature 473 (2011): 101–104. 21471965 10.1038/nature09881 PMC3088778

[15] K. A. Kwei , Y. H. Kim , L. Girard , et al., “Genomic Profiling Identifies TITF1 as a Lineage‐Specific Oncogene Amplified in Lung Cancer,” Oncogene 27 (2008): 3635–3640. 18212743 10.1038/sj.onc.1211012 PMC2903002

[16] N. Frost , T. Zhamurashvili , M. von Laffert , et al., “Pemetrexed‐Based Chemotherapy Is Inferior to Pemetrexed‐Free Regimens in Thyroid Transcription Factor 1 (TTF‐1)‐negative, EGFR/ALK‐Negative Lung Adenocarcinoma: A Propensity Score Matched Pairs Analysis,” Clinical Lung Cancer 21 (2020): e607–e621. 32620471 10.1016/j.cllc.2020.05.014

[17] J. B. Schilsky , A. Ni , L. Ahn , et al., “Prognostic Impact of TTF‐1 Expression in Patients With Stage IV Lung Adenocarcinomas,” Lung Cancer 108 (2017): 205–211. 28625636 10.1016/j.lungcan.2017.03.015 PMC6423973

[18] M. K. Doherty , E. O'Connor , D. Hannon , et al., “Absence of Thyroid Transcription Factor‐1 Expression Is Associated With Poor Survival in Patients With Advanced Pulmonary Adenocarcinoma Treated With Pemetrexed‐Based Chemotherapy,” Irish Journal of Medical Science 188 (2019): 69–74. 29948461 10.1007/s11845-018-1839-5

[19] O. Fiala , M. Pesek , J. Skrickova , et al., “Thyroid Transcription Factor 1 Expression Is Associated With Outcome of Patients With Non‐Squamous Non‐Small Cell Lung Cancer Treated With Pemetrexed‐Based Chemotherapy,” Tumour Biology 39 (2017): 1010428317691186. 28218046 10.1177/1010428317691186

[20] L. Brunetti , V. Santo , A. Galletti , et al., “TTF‐1 Negativity Predicts Poor Outcomes in Advanced Non‐Squamous NSCLC Also in the Immunotherapy Era: A Multicenter Cohort Study and Meta‐Analysis,” Cancers 17 (2025): 2188. 40647486 10.3390/cancers17132188 PMC12248803

[21] Y. Shiraishi , T. Shimose , M. Tachihara , et al., “Atezolizumab With Carboplatin Plus Nab‐Paclitaxel Combination Therapy for TTF‐1 Negative Advanced Nonsquamous Non‐Small Cell Lung Cancer: A Multicenter, Single‐Arm Phase 2 Trial (F1NE TUNE, LOGiK 2102),” European Journal of Cancer 229 (2025): 115779. 40961811 10.1016/j.ejca.2025.115779

[22] A. Jeanson , P. Tomasini , M. Souquet‐Bressand , et al., “Efficacy of Immune Checkpoint Inhibitors in KRAS‐Mutant Non‐Small Cell Lung Cancer (NSCLC),” Journal of Thoracic Oncology 14 (2019): 1095–1101. 30738221 10.1016/j.jtho.2019.01.011

[23] S. C. M. Lau , A. F. Fares , L. W. Le , et al., “Subtypes of EGFR‐ and HER2‐Mutant Metastatic NSCLC Influence Response to Immune Checkpoint Inhibitors,” Clinical Lung Cancer 22 (2021): 253–259. 33582070 10.1016/j.cllc.2020.12.015

[24] A. G. Nicholson , M. S. Tsao , M. B. Beasley , et al., “The 2021 WHO Classification of Lung Tumors: Impact of Advances Since 2015,” Journal of Thoracic Oncology 17 (2022): 362–387. 34808341 10.1016/j.jtho.2021.11.003

[25] M. Reck , D. Rodríguez‐Abreu , A. G. Robinson , et al., “Updated Analysis of KEYNOTE‐024: Pembrolizumab Versus Platinum‐Based Chemotherapy for Advanced Non‐Small‐Cell Lung Cancer With PD‐L1 Tumor Proportion Score of 50% or Greater,” Journal of Clinical Oncology 37 (2019): 537–546. 30620668 10.1200/JCO.18.00149

[26] K. Morimoto , J. Uchino , T. Yokoi , et al., “Early Discontinuation of Induction Therapy in Chemoimmunotherapy as an Effective Alternative to the Standard Regimen in Patients With Non‐Small Cell Lung Cancer: A Retrospective Study,” Journal of Cancer Research and Clinical Oncology 148 (2022): 2437–2446. 34510271 10.1007/s00432-021-03782-5 PMC11801065

[27] N. Nishioka , H. Kawachi , T. Yamada , et al., “Unraveling the Influence of TTF‐1 Expression on Immunotherapy Outcomes in PD‐L1‐High Non‐Squamous NSCLC: A Retrospective Multicenter Study,” Frontiers in Immunology 15 (2024): 1399889. 39076994 10.3389/fimmu.2024.1399889 PMC11284020

[28] N. Nishioka , T. Hata , T. Yamada , et al., “Impact of TTF‐1 Expression on the Prognostic Prediction of Patients With Non‐Small Cell Lung Cancer With PD‐L1 Expression Levels of 1% to 49%, Treated With Chemotherapy vs. Chemoimmunotherapy: A Multicenter, Retrospective Study,” Cancer Research and Treatment 57 (2025): 412–421. 39482992 10.4143/crt.2024.748 PMC12016825

[29] M. A. Socinski , R. M. Jotte , F. Cappuzzo , et al., “Atezolizumab for First‐Line Treatment of Metastatic Nonsquamous NSCLC,” New England Journal of Medicine 378 (2018): 2288–2301. 29863955 10.1056/NEJMoa1716948

[30] L. Paz‐Ares , T. E. Ciuleanu , M. Cobo , et al., “First‐Line Nivolumab Plus Ipilimumab Combined With Two Cycles of Chemotherapy in Patients With Non‐Small‐Cell Lung Cancer (CheckMate 9LA): An International, Randomised, Open‐Label, Phase 3 Trial,” Lancet Oncology 22 (2021): 198–211. 33476593 10.1016/S1470-2045(20)30641-0

[31] M. L. Johnson , B. C. Cho , A. Luft , et al., “Durvalumab With or Without Tremelimumab in Combination With Chemotherapy as First‐Line Therapy for Metastatic Non‐Small‐Cell Lung Cancer: The Phase III POSEIDON Study,” Journal of Clinical Oncology 41 (2023): 1213–1227. 36327426 10.1200/JCO.22.00975 PMC9937097

[32] M. C. Garassino , S. Gadgeel , G. Speranza , et al., “Pembrolizumab Plus Pemetrexed and Platinum in Nonsquamous Non‐Small‐Cell Lung Cancer: 5‐Year Outcomes From the Phase 3 KEYNOTE‐189 Study,” Journal of Clinical Oncology 41 (2023): 1992–1998. 36809080 10.1200/JCO.22.01989 PMC10082311

[33] M. Nishio , S. Watanabe , H. Udagawa , et al., “Integrated Analysis of Older Adults and Patients With Renal Dysfunction in the IMpower130 and IMpower132 Randomized Controlled Trials for Advanced Non‐Squamous Non‐Small Cell Lung Cancer,” Lung Cancer 196 (2024): 107859. 39127586 10.1016/j.lungcan.2024.107859

[34] J. D. Patel , M. A. Socinski , E. B. Garon , et al., “PointBreak: A Randomized Phase III Study of Pemetrexed Plus Carboplatin and Bevacizumab Followed by Maintenance Pemetrexed and Bevacizumab Versus Paclitaxel Plus Carboplatin and Bevacizumab Followed by Maintenance Bevacizumab in Patients With Stage IIIB or IV Nonsquamous Non‐Small‐Cell Lung Cancer,” Journal of Clinical Oncology 31 (2013): 4349–4357. 24145346 10.1200/JCO.2012.47.9626 PMC4881367

[35] J. Yao , S. Li , L. Bai , et al., “Efficacy and Safety of Immune Checkpoint Inhibitors in Elderly Patients With Advanced Non‐Small Cell Lung Cancer: A Systematic Review and Meta‐Analysis,” EClinicalMedicine 81 (2025): 103081. 39975700 10.1016/j.eclinm.2025.103081 PMC11836518

[36] J. Yin , Y. Song , Y. Fu , et al., “The Efficacy of Immune Checkpoint Inhibitors Is Limited in Elderly NSCLC: A Retrospective Efficacy Study and Meta‐Analysis,” Aging 15 (2023): 15025–15049. 38127004 10.18632/aging.205328 PMC10781456

[37] J. V. Alessi , B. Ricciuti , E. Jiménez‐Aguilar , et al., “Outcomes to First‐Line Pembrolizumab in Patients With PD‐L1‐High (≥50%) Non‐Small Cell Lung Cancer and a Poor Performance Status,” Journal for Immunotherapy of Cancer 8 (2020): e001007. 32753547 10.1136/jitc-2020-001007 PMC7406027

[38] R. Zinner , C. Visseren‐Grul , D. R. Spigel , and C. Obasaju , “Pemetrexed Clinical Studies in Performance Status 2 Patients With Non‐Small Cell Lung Cancer (Review) (Review),” International Journal of Oncology 48 (2016): 13–27. 26530033 10.3892/ijo.2015.3219 PMC4734604

[39] L. Righi , T. Vavalà , I. Rapa , et al., “Impact of Non‐Small‐Cell Lung Cancer‐Not Otherwise Specified Immunophenotyping on Treatment Outcome,” Journal of Thoracic Oncology 9 (2014): 1540–1546. 25521399 10.1097/JTO.0000000000000271

[40] Y. Katayama , T. Yamada , K. Morimoto , et al., “TTF‐1 Expression and Clinical Outcomes of Combined Chemoimmunotherapy in Patients With Advanced Lung Adenocarcinoma: A Prospective Observational Study,” JTO Clinical and Research Reports 4 (2023): 100494. 37020925 10.1016/j.jtocrr.2023.100494 PMC10067944

[41] S. Mori , T. Maiguma , K. Yoshii , et al., “Effect of the Thyroid Transcription Factor 1 Expression and Treatment Discontinuation due to Adverse Events on Progression‐Free Survival in Patients With Advanced Non‐Squamous Non‐Small Cell Lung Cancer Treated With Pembrolizumab Plus Pemetrexed and Platinum Chemotherapy: A Japanese Four‐Hospital, Retrospective Study,” American Journal of Cancer Research 14 (2024): 3852–3858. 39267683 10.62347/JTWP3747 PMC11387863

[42] R. Ibusuki , Y. Yoneshima , M. Hashisako , et al., “Association of Thyroid Transcription Factor‐1 (TTF‐1) Expression With Efficacy of PD‐1/PD‐L1 Inhibitors Plus Pemetrexed and Platinum Chemotherapy in Advanced Non‐Squamous Non‐Small Cell Lung Cancer,” Translational Lung Cancer Research 11 (2022): 2208–2215. 36519019 10.21037/tlcr-22-393 PMC9742625

[43] F. Skoulidis , L. A. Byers , L. Diao , et al., “Co‐Occurring Genomic Alterations Define Major Subsets of KRAS‐Mutant Lung Adenocarcinoma With Distinct Biology, Immune Profiles, and Therapeutic Vulnerabilities,” Cancer Discovery 5 (2015): 860–877. 26069186 10.1158/2159-8290.CD-14-1236 PMC4527963

[44] F. Skoulidis , B. T. Li , A. J. de Langen , et al., “Molecular Determinants of Sotorasib Clinical Efficacy in KRAS^G12C^‐Mutated Non‐Small‐Cell Lung Cancer,” Nature Medicine 31 (2025): 2755–2767. 10.1038/s41591-025-03732-5 PMC12353874 40437272

[45] L. Sun , E. A. Handorf , Y. Zhou , H. Borghaei , C. Aggarwal , and J. Bauman , “Outcomes in Patients Treated With Frontline Immune Checkpoint Inhibition (ICI) for Advanced NSCLC With KRAS Mutations and STK11/KEAP1 Comutations Across PD‐L1 Levels,” Lung Cancer 190 (2024): 107510. 38432028 10.1016/j.lungcan.2024.107510 PMC11194721

[46] F. Skoulidis , M. E. Goldberg , D. M. Greenawalt , et al., “ *STK11/LKB1* Mutations and PD‐1 Inhibitor Resistance in *KRAS*‐Mutant Lung Adenocarcinoma,” Cancer Discovery 8 (2018): 822–835. 29773717 10.1158/2159-8290.CD-18-0099 PMC6030433

[47] D. J. Cantor , H. Nimeiri , L. Horn , et al., “Outcomes Following First‐Line Immune Checkpoint Inhibitors With or Without Chemotherapy Stratified by KRAS Mutational Status‐A Real‐World Analysis in Patients With Advanced NSCLC,” Clinical Lung Cancer 26 (2025): 503–510.e4. 40544018 10.1016/j.cllc.2025.05.007

[48] A. J. Cooper , A. Muzikansky , J. Lennerz , et al., “Clinicopathologic Characteristics and Outcomes for Patients With *KRAS* G12D‐Mutant NSCLC,” JTO Clinical and Research Reports 3 (2022): 100390. 36118132 10.1016/j.jtocrr.2022.100390 PMC9471201

[49] F. Skoulidis , H. A. Araujo , M. T. Do , et al., “CTLA4 Blockade Abrogates KEAP1/STK11‐Related Resistance to PD‐(L)1 Inhibitors,” Nature 635 (2024): 462–471. 39385035 10.1038/s41586-024-07943-7 PMC11560846

[50] R. J. G. Cardnell , C. Behrens , L. Diao , et al., “An Integrated Molecular Analysis of Lung Adenocarcinomas Identifies Potential Therapeutic Targets Among TTF1‐Negative Tumors, Including DNA Repair Proteins and Nrf2,” Clinical Cancer Research 21 (2015): 3480–3491. 25878335 10.1158/1078-0432.CCR-14-3286 PMC4526428

[51] W. Cheng , B. Xu , H. Zhang , and S. Fang , “Lung Adenocarcinoma Patients With KEAP1 Mutation Harboring Low Immune Cell Infiltration and Low Activity of Immune Environment,” Thoracic Cancer 12 (2021): 2458–2467. 34328274 10.1111/1759-7714.14089 PMC8447911

[52] S. K. Karthikeyan , N. T. Gimbrone , T. R. Percy , and W. D. Cress , “Loss of Cellular Identity in Common Pre‐Clinical Models of Serine‐Threonine Kinase 11 (Liver Kinase B1) Loss,” Cancer Treatment and Research Communications 26 (2021): 100286. 33338855 10.1016/j.ctarc.2020.100286 PMC10022640

[53] D. Rodríguez‐Abreu , S. F. Powell , M. J. Hochmair , et al., “Pemetrexed Plus Platinum With or Without Pembrolizumab in Patients With Previously Untreated Metastatic Nonsquamous NSCLC: Protocol‐Specified Final Analysis From KEYNOTE‐189,” Annals of Oncology 32 (2021): 881–895. 33894335 10.1016/j.annonc.2021.04.008

[54] D. Fujimoto , S. Miura , K. Yoshimura , et al., “A Real‐World Study on the Effectiveness and Safety of Pembrolizumab Plus Chemotherapy for Nonsquamous NSCLC,” JTO Clinical and Research Reports 3 (2021): 100265. 35146460 10.1016/j.jtocrr.2021.100265 PMC8819387

[55] J. R. Brahmer , C. Lacchetti , B. J. Schneider , et al., “Management of Immune‐Related Adverse Events in Patients Treated With Immune Checkpoint Inhibitor Therapy: American Society of Clinical Oncology Clinical Practice Guideline,” Journal of Clinical Oncology 36 (2018): 1714–1768. 29442540 10.1200/JCO.2017.77.6385 PMC6481621

[56] X. Zhou , Z. Yao , H. Yang , N. Liang , X. Zhang , and F. Zhang , “Are Immune‐Related Adverse Events Associated With the Efficacy of Immune Checkpoint Inhibitors in Patients With Cancer? A Systematic Review and Meta‐Analysis,” BMC Medicine 18 (2020): 87. 32306958 10.1186/s12916-020-01549-2 PMC7169020

[57] B. Shankar , J. Zhang , A. R. Naqash , et al., “Multisystem Immune‐Related Adverse Events Associated With Immune Checkpoint Inhibitors for Treatment of Non‐Small Cell Lung Cancer,” JAMA Oncology 6 (2020): 1952–1956. 33119034 10.1001/jamaoncol.2020.5012 PMC7596677

[58] M. A. Socinski , R. M. Jotte , F. Cappuzzo , et al., “Association of Immune‐Related Adverse Events With Efficacy of Atezolizumab in Patients With Non‐Small Cell Lung Cancer: Pooled Analyses of the Phase 3 IMpower130, IMpower132, and IMpower150 Randomized Clinical Trials,” JAMA Oncology 9 (2023): 527–535. 36795388 10.1001/jamaoncol.2022.7711 PMC9936386

[59] N. Frost , A. Bleckmann , F. Griesinger , et al., “Rationale and Design of the Phase II ANTELOPE Study of Atezolizumab, Carboplatin and Nab‐Paclitaxel vs. Pembrolizumab, Platinum and Pemetrexed in TTF‐1 Negative, Metastatic Lung Adenocarcinoma (AIO‐TRK‐0122),” Clinical Lung Cancer 24 (2023): 568–572. 37169628 10.1016/j.cllc.2023.04.009

[60] M. Tachihara , T. Miyawaki , Y. Sato , et al., “P3.18.31 Phase II Study of CBDCA + Nab‐PTX + Tremelimumab+Durvalumab for TTF‐1 Negative Advanced Non‐Sq NSCLC (WJOG17223L, TURNING),” Journal of Thoracic Oncology 20 (2025): S558.

